# What can be learnt from a snail?

**DOI:** 10.1111/eva.12277

**Published:** 2015-07-07

**Authors:** Kerstin Johannesson

**Affiliations:** ^1^ Department of Marine Sciences ‐ TjärnöUniversity of GothenburgStrömstadSweden

**Keywords:** ecotypes, *Littorina saxatilis*, parallel evolution, primary hybrid zones, speciation with gene flow

## Abstract

The marine snail *Littorina saxatilis* is a common inhabitant of intertidal shores of the north Atlantic. It is amazingly polymorphic and forms reproductively isolated ecotypes in microhabitats where crabs are either present and wave action is less furious, or where waves are strong and crabs are absent. Decades of research have unveiled much of the ecological and demographic context of the formation of crab‐ and wave‐ecotype snails showing important phenotypic differences being inherited, differential selection being strong over adjacent microhabitats, local dispersal being restricted, and long‐distance transports of individuals being rare. In addition, strong assortative mating of ecotypes has been shown to include a component of male mate preference based on female size. Several studies support ecotypes being diverged locally and under gene flow in a parallel and highly replicated fashion. The high level of replication at various levels of independence (from local to pan‐European scale) provides excellent opportunities to investigate the detailed mechanisms of microevolution, including the formation of barriers to gene flow. Current investigations benefit from a draft reference genome and an integration of genomic approaches, modelling and experiments to unveil molecular and ecological components of speciation and their interactions.

## Introduction

It is somewhat fascinating that in evolution we are still investigating the conceptual ideas that Darwin and his peers raised and discussed already 150 years ago. However, we are now in the middle of a very exciting time when most biological research is profiting from new technologies (next‐generation sequencing, computer‐based data analyses and modelling), and these technologies will throw much new light on many of the classical questions that, by and large, remain the same. One such question is how new biodiversity, and in particular new species, is formed (the ‘mystery of mysteries’).

It comes as no surprise that many studies of speciation have started as an attempt to resolve a messy taxonomy of a group of closely related species, and now many of these groups are objects for studies of speciation and its mechanisms (reviewed in Seehausen et al. 2015). Here, I will describe one such case. Starting from a confused taxonomy, the marine snail *Littorina saxatilis* has become an important model for evolutionary studies. As I have myself been heavily involved in this development, the review of this system will also be a somewhat egocentric journey through much of my own scientific career. A journey that has also given me some personal experiences with relevance to the discussion of gender in science (Box 1).

Box 1 **Female in science – my perspective**

My personal experience of being a female in a male‐dominated scientific discipline is actually very positive. I have throughout my career being treated on equal basis as my male colleagues, and even, in some cases, been favoured for the sake of equality in decision of funding or positions in grant committees. My own involvement in collaborations and networks has also worked out well, even so most of my colleagues have been men, including acceptance (and perhaps also appreciation) of me acting director and PI in large research programs. How come that my experience is so different from other female colleagues? The most important factor is probably how you are treated by your mentor and senior colleagues. In my case, I did not have a mentor or supervisor during PhD and postdoc training, so I was free to choose to work with any collaborator, and I found a couple of very skilled and inspiring, somewhat more senior colleagues that helped me a lot during my initial years (Dave Raffaelli and Bob Ward, to mention the two most important ones). Moreover the former director of the Tjärnö marine laboratory where I ‘grew up’, Lars Afzelius, was a person that supported young scientists and early gave me both responsibilities and credits. Perhaps, reassurance and positive reinforcement are the most importance components that a young scientist (independent of gender) need for a positive development in science.

As a mature female scientist, I have experienced some gender‐related differences that are, however, not necessarily negative. For example, I am more frequently than my male colleagues asked to sit in various evaluation committees, often to fill a quota of the underrepresented gender. This is good scientific training but also beneficial for networking, although time consuming. Furthermore, I have attracted more female students and also more female collaborators than many of my male colleagues, and this leads to gender balance (good for interactions in a group) and to less criticism for gender inequality from evaluating panels.

Thus, my conclusion is that today (at least in Sweden) gender equality problems are strongly context related, and what can negatively affect a young female scientist can as well happen to a young male scientist. Consequently, my advice to any young scientist is to find an open research environment and a mentor that give positive support, and thereafter choose among available topics to work with, remembering that the most important driver in research is to have fun at work.

## In the wake of a confused *Littorina* taxonomy

**Figure 1:**
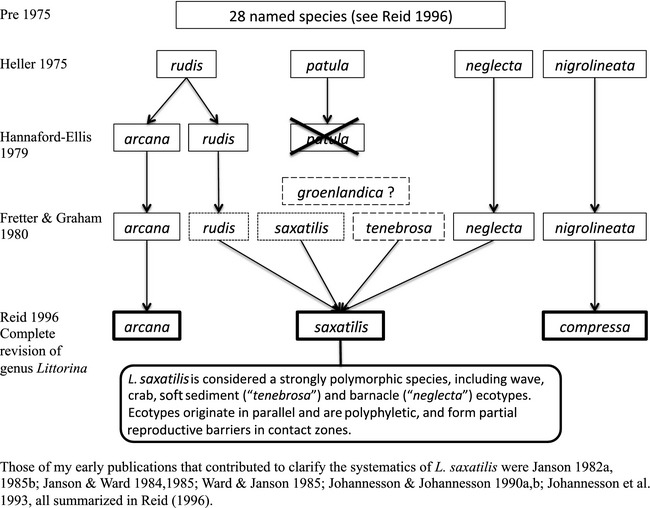
Modern taxonomic revisions of the *Littorina saxatilis* species complex. (For a comprehensive review of the taxonomy of the genus *Littorina* see Reid 1996.) Those of my early publications that contributed to clarify the systematics of *L. saxatilis* were Janson (1982a, 1985b); Janson and Ward (1984, 1985); Ward and Janson (1985); Johannesson and Johannesson (1990a,b); Johannesson et al. 1993; all summarized in Reid (1996).

Up until the late 70s, evolution and systematics of wild organisms were mainly studied using phenotypic traits. This was also the case for species of marine intertidal periwinkles of the genus *Littorina* (Sacchi and Rastelli 1966; James 1968; Heller 1975; Fretter and Graham 1980). Despite the many systematic investigations of this genus, the taxonomy of *Littorina* remained confused, and this was particularly true for the species, today named *L. saxatilis* (Fig. 1). The problem emerged from an unusually strong polymorphism that messed up classical shell traits commonly used in gastropod taxonomy. Earlier taxonomic work had, based on shell shape and colours, resulted in the species, we today refer to as one species, *L. saxatilis*, being described under 28 different species names, in addition to named colour and shape varieties (Reid 1996). In addition, a young female graduate student had suggested the presence of a new species (*L. arcana*, Hannaford‐Ellis 1979) that could not be discriminated using shell traits, and which had been overlooked by experienced mollusc taxonomists for decades. All this was annoying to the scientific community, while also an interesting feature in itself that asked for its evolutionary explanations.

In 1978, I was looking for a topic for a master project and Anders Warén suggested to me to do a taxonomic description of two morphs of *L. saxatilis* found on the Swedish west coast that he thought were separate species. The objective seemed straight forward, but soon it became obvious to me that morphological intermediates between the two supposed species were frequent in particular sites. I mentioned this dilemma to Anders Warén. ‘OK’, he said, ‘then it must be sympatric speciation’. What a scientific intuition! But it took us another 35 years to find thorough support for this suggestion (Butlin et al. 2014).

**Figure 2:**
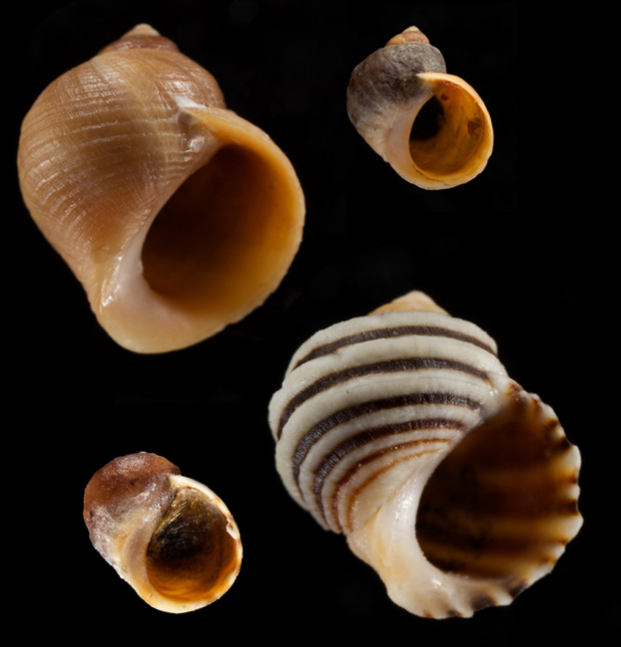
Swedish crab ecotype (top left) and wave ecotype (top right), and Spanish wave ecotype (bottom left) and crab ecotype (bottom right). A morphometric analysis of shape similarities is given in Butlin et al. (2014). Photo: Fredrik Pleijel.

My master topic thus failed from the start, and I had to redirect the project towards a more evolutionary question, asking why such a strong polymorphism had evolved and how. At this time I did not realize that this was a lifetime project rather than a master study. My master thesis thus presented a morphological description of two distinct ecotypes of *L. saxatilis* strongly linked to separate microhabitats along the Swedish rocky shores (Janson 1982a). In addition, I described the presence of snails of intermediate morphology appearing over sharp environmental ecotones where the two ecotypes formed contact zones. One ecotype was present in boulder shores where crabs are abundant, and where snails are protected from direct wave forces by hiding back and under boulders. This ecotype I named ‘sheltered (S) ecotype’, but later we have renamed it to ‘crab ecotype’ (Johannesson et al. 2010a). The other ecotype was confined to wave‐swept rocky surfaces devoid of crabs and initially named ‘exposed (E) ecotype’ or later ‘wave ecotype’. The adult crab ecotype is large and robust with thick shell, relatively small aperture and a high spire, while the adult wave ecotype is on average half the size of the crab ecotype, has a thin shell, a relatively large aperture and a low spire (Fig. 2). In contact zones, snail morphologies represent a continuum from one morph to the other, with all possible intermediate stages (Janson and Sundberg 1983).

## Local adaption of ecotypes

Neglecting a professor's advice to ‘choose a group of organisms for which not all interesting studies have already been performed’, I started up a PhD project with a first aim to look for postzygotic barriers. If present, this would suggest that the two ecotypes had an allopatric origin and that formation of intermediates was a result of hybridization following secondary contact. However, females from contact zones were as fecund and fertile as other females (Janson 1985a). Moreover, snails of intermediate morphologies had a relatively high fitness in the contact zone compared to individuals of the two pure ecotypes, while selected against in both crab and wave environments (Janson 1983a), suggesting reinforcement of mating barriers being less of a driver. In another intertidal gastropod species, *Nucella lapillus*, two chromosomal races had been found (Bantock and Cockayne 1975), somewhat associated with different microenvironments, but both ecotypes of *L. saxatilis* had the same chromosomal number and similar caryotypes (Janson 1983b).

**Figure 3:**
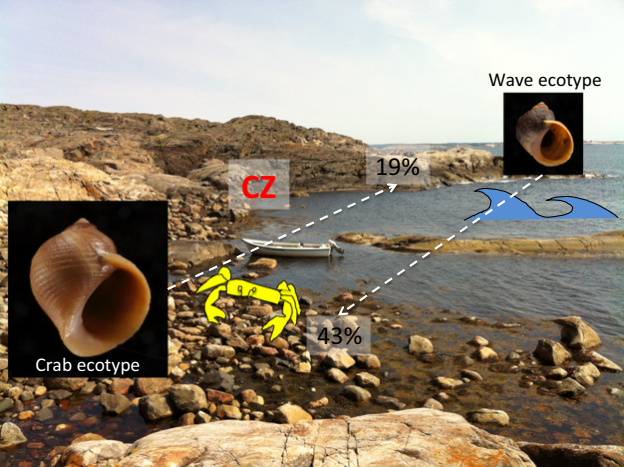
A Swedish rocky shore divided into crab and wave microenvironments, and survival of transplanted individuals of *Littorina saxatilis* crab and wave ecotypes compared to that of nontransplanted individuals. Mean dispersal distances in their native environments are 2.1 m per 3 months in the crab ecotype and 1.1 m per 3 month in the wave ecotype. CZ indicates the position of the ~20 m wide contact zone of crab and wave microenvironments in which selection pressures are mixed. (Data from Janson 1983a averaged over four different seasons.)

*Littorina saxatilis* is a direct developer releasing 0.5‐mm‐sized crawl‐away juveniles directly on the shore (instead of broadcasting pelagic larva that most marine gastropods do), and I soon realized that juvenile and adult migration, together with local selection pressures, must be key components in local adaptation of ecotypes. In an attempt to assess these components, I moved marked individuals from one habitat to the other and used controls moved within habitats, and I also repeated the experiment over four different seasons. The results showed strong phenotypic selection, restricted migration and inherited effects of growth being stronger than environmental effects, with some variation over seasons. For example, I found that moving snails between microhabitats (a translocation of only about 50 m) decreased the probability of survival to about 20–45% of what it was in the native microhabitat and more so for adult than for juvenile snails (Fig. 3). Furthermore, most snails were very stationary and did not move more than one or a few metres during 3 months of periods even after transplanted to a foreign habitat (Janson 1983a). Moving them between habitats somewhat affected their growth rate, but the faster growth rate of the crab ecotype and the slower growth rate of the wave ecotype persisted after being transplanted to a contrasting habitat (Janson 1982b).

Later, and in collaboration with Emilio Rolán‐Alvarez from Vigo, we made similar reciprocal transplantations in Spanish populations of crab and wave ecotype *L. saxatilis* (Fig. 2). Here, the two ecotypes are vertically distributed with the crab ecotype in the upper shore and the wave ecotype in the lower shore, and an extended zone of hybridization in between. Despite the very different local context, we found much the same results in Spain as in Sweden. That is, snails of one ecotype survived much better in their native microhabitat than if moved to the other microhabitat (Rolán‐Alvarez et al. 1997). In addition, snails did not migrate out of their microhabitat, but if moved to the other habitat they showed a tendency of returning to their intertidal zone (Erlandsson et al. 1998). Also in Spain, snails of the wave ecotype grew more slowly than the crab ecotype in both habitats, again suggesting inherited components of growth rate (Johannesson et al. 1997).

We also raised Swedish snails of different ecotypes in a common garden and found that a dominant part of the phenotypic differences in nature were indeed maintained in the next, laboratory‐raised, generation (Johannesson and Johannesson 1996), results that seem robust over several generations of common garden experiment (K. Johannesson, personal observation). Experimental trials by Johan Hollander have, however, also showed that some plasticity adds to the adaptive differences, in a direction so that they further support the local survival of ecotypes to their native microenvironment (Hollander et al. 2006; Hollander and Butlin 2010).

Interestingly, ecotype characteristics also include different behaviours (snail ‘personalities’) with the wave ecotype being reluctant to withdraw into the shell while the crab ecotype is very quick to go inside following only slight disturbance (Johannesson and Johannesson 1996). These differences are inherited and likely to be adaptive in the two microenvironments as being under differential selection. Indeed, in the wave‐exposed environment, it is important to remain attached to the substratum and disturbed snails should soon emerge from the shell to reattach. In a crab‐rich environment, it is instead beneficial to quickly retract into the shell and remain there if disturbed.

The relationship between crab predation and shell shape was further investigated by Bo Johannesson (1986), and he found that crabs used either crushing or peeling to get snails out of the shell, and therefore both a strong shell and a relatively small aperture (characteristic of snails in crab environments) protected snails from crab attacks. While it seems a qualified guess that the large aperture and foot of the wave ecotype improve attachment strength to the substratum under wave action, this is technically challenging to investigate. However, some simple measurements showed that in a water flow of 1.2 m per second 50% of attached snails loose their grip (Johannesson et al. 2010b), and wave‐ecotype snails resist better high water speed than do crab‐ecotype snails (Rolán‐Alvarez et al. 1997).

Although the wave and crab ecotypes have been the focus for most of the studies of *L. saxatilis*, it is important to notice that additional ecotypes can be found, including snails that appear more or less intermediate to ‘named’ ecotypes (Reid 1996). In fact, it seems as whenever the species has established in a microhabitat with a specific combination of different biotic and abiotic stressors, a new ecotype is formed. In Galicia (NW Spain) and along the Swedish west coast, the crab and wave ecotypes are predominant due to the subdivision of most shores into either crab‐rich or wave‐swept microenvironments. In addition, the crab and wave ecotypes have been found in some areas in the UK (e.g. Grahame et al. 2006). In addition to size and shape variation, shell colour variation is extensive and complex in this species and seems to have both a local and a regional component, and being influenced by both selection and genetic drift (Ekendahl and Johannesson 1997; Johannesson and Ekendahl 2002).

## Genetic structure of *L. saxatilis*

What is, perhaps, most surprising with the division into crab and wave ecotypes of *L. saxatilis* in Spain, Sweden and the UK, is that even small patches of local habitat seems enough to promote a population of a distinct ecotype. The restricted dispersal and very strong divergent selection seems to contribute to maintain local adaptation at spatial scales down to a few metres of shore. But what genomic architecture may give rise to such a fine‐scaled population structure, and from where comes the genetic variation and is it organized in a highly structured way? Essentially, two main hypotheses would explain this pattern. Firstly, different ecotypes may be of separate origin and may have evolved differently under an earlier period of allopatry, perhaps isolated in separate refugia during a glaciation period. Thereafter, they spread into secondary overlap under which they started to hybridize. An alternative hypothesis is that the two ecotypes formed *in situ* in each island, or local site, after the initial colonization of the local site by one of the ecotypes, or by some more intermediate phenotype. The second hypothesis implicitly involves repeatability, at some spatial level, but not necessarily at the smallest spatial scale (such as a local site or a small island).

Although, my thoughts about these two alternatives matured in a discussion with colleagues much later (Johannesson et al. 2010a), I made some early attempts to bring light upon the genetic relationship between populations of the different ecotypes, and how this related to the geographic separation of populations. To avoid too much of supposed geographic isolation effects, the first investigation was focused on samples of different ecotypes from microhabitats in a small area. This study was performed with a very large involvement of Bob Ward who had introduced allozyme electrophoresis to *Littorina* studies a few years earlier (Ward and Warwick 1980). Thus, we run allozyme analyses of 11 samples of snails from alternating crab and wave microhabitats along a 1‐km shore in a Swedish island, including samples from a few hybrid zones. The results were striking. First of all, we found an unexpectedly high level of overall genetic differentiation, with *F*_ST_ values similar in magnitude to what was known at that time as the level of differentiation among human populations on a global scale (Janson and Ward 1984). An even more interesting finding was that despite the high genetic differentiation among samples, this variation was in most loci uncorrelated with ecotype, which led us to conclude that the two ecotypes were not well separated as subspecies (or species).

Allozyme electrophoresis was, in the early 80s, a powerful tool to get insights on genetic structure, gene flow and potentially selection, and together with Bob Ward I contributed to sort out some of the taxonomic issues of the *L. saxatilis* complex (see Fig. 1). In addition, I tested the effect of geographic isolation and founder events on the genetic structure of *L. saxatilis*. Thus, in a comparison with a closely related species with broadcasting larvae (*L. littorea*) I found very strong isolation by distance effects in *L. saxatilis* while no differentiation at all in *L. littorea* over a distance of 500 km (Janson 1987a). This highlighted the importance of a direct development for the population genetic structure of *L. saxatilis*. In the absence of pelagic larvae, one would also expect that colonization of empty habitats would be rather occasional in *L. saxatilis* and probably undertaken by small founder groups. Finding lower levels of heterozygosity in populations of snails living on small and recently populated postglacial islands compared to heterozygosity of mainland populations led to the conclusion that indeed, founder effects were present in some populations (Janson 1987b).

## Molecular variation under strong selection

**Figure 4:**
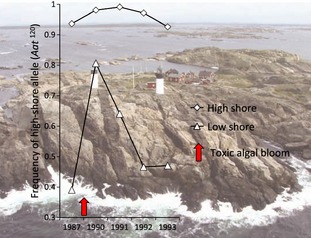
Changes in the frequency of the high‐shore allele of aspartate aminotransferase (*Aat* ^120^) in a population of *L. saxatilis* in the Swedish island Ursholmen between 1987 and 1993. Throughout NW Europe, *Aat* ^120^ dominates high shores and *Aat* ^100^ low shores (Johannesson and Johannesson 1989). Complete extinction of low‐shore snails in 1988 by a unique toxic algal bloom was followed by colonization of the low‐shore by high‐shore snails resulting in increased frequencies of *Aat* ^120^ in the low shore. However, strong directional selection pushed frequencies back to pre‐1988 after 5–6 snail generations. (Data from Johannesson et al. 1995a).

Intertidal habitats have strong vertical gradients in many physical parameters and to find out if the genetic variation in *L. saxatilis* was to any extent affected by such gradients, we analysed allozyme variation in samples of wave ecotype over vertical shore gradients of 5–6 m (low‐shore to high‐shore splash zone) in heavily exposed rocky shores in Sweden, the UK, Iceland and Norway. While four strongly polymorphic allozyme loci did not vary much over these transects, we found strong allele frequency clines in the aspartate aminotransferase (*Aat*) locus with a dominance of one allele (*Aat* ^100^) in all low‐shore samples and a dominance of another allele (*Aat* ^120^) in all high‐shore samples from the different countries (Johannesson and Johannesson 1989). Lucky enough (!) a year later the Swedish west coast and two of the earlier sampled *Aat*‐clines were hit by a toxic algal bloom that killed all *L. saxatilis* living in the lower part of shores. The years that followed, snails from the high shore (mostly homozygote for the *Aat* ^120^ allele) recolonized the low shore that before the algal bloom was dominated by homozygotes of the *Aat* ^100^ allele. Following down migration of snails with *Aat* ^120^ alleles, the steep allelic cline disappeared, but re‐established after only 5–6 generations due to strong differential selection on the *Aat* locus, or on a very closely linked locus (Fig. 4). The rate of the transformation was remarkable fast and strength of selection estimated to be very high (*s* = 0.4) (Johannesson et al. 1995a). The parallel clines in geographically spread areas, along with enzyme activity measurements (Panova and Johannesson 2004), strongly suggested selection acting directly on the *Aat* locus, and ongoing studies now show two nonsynonymous mutations most likely being involved in the fitness differences of the two allozymes.

The strong isolation by distance that structure the allozyme variation of the species (Janson and Ward 1984; Janson 1987a) is thus complemented with genetic divergence caused by differential selection over contrasting microhabitats, in at least some loci. In an attempt to separate the two factors generating variation (habitat‐linked differential selection and genetic drift caused by geographic isolation) Andrey Tatarenkov, who came to my laboratory from Vladivostok, found a way to modify the classical hierarchical amova into an orthogonal amova with which the genetic variation among samples could be separated into variation among islands (presumably caused by genetic drift), and variation between habitats (presumably a result of differential selection), in addition to the interaction of the two factors. Sampling a number of islands, and both wave and crab microhabitats in a small area on the Swedish west coast, we found not only significant divergence among island populations in almost all allozyme loci, but in addition, habitat‐linked divergence in several loci (including *Aat*) (Johannesson and Tatarenkov 1997). This suggested that not only phenotypic traits are under strong habitat‐linked selection in this species, but also that variation in several allozyme loci is maintained by balancing selection.

## Dispersal and colonization

*Littorina saxatilis* is geographically widespread and found from Greenland, Iceland and Svalbard in the north to Gibraltar and Virginia in the south (Reid 1996). In postglacial areas, such as the Swedish west coast, main habitats are small islands and rocks brought to the surface by postglacial land lifts. Notably, it is still unknown how new (e.g. postglacial) habitats were colonized by the species after the retreat of the ice, but phylogeographic data show that the species survived the last glacial period in refugia at northern latitudes and were able to maintain both a large area of distribution and large population size during this period of time (Panova et al. 2011).

Rafting has been suggested as a possible, although very occasional, spreading mechanism of single snails, but quantifying occasional events like rafting is not easy. However, following the toxic algal bloom in 1988 that completely wiped out small populations living on small shallow islets (skerries) in the Swedish archipelago, we followed the recolonization of these habitats and found that, on average, 3% of all skerry populations were re‐established per generation (Johannesson and Johannesson 1995). This was a surprisingly high rate of colonization of empty habitats for a species that has no swimming ability during larval or adult stage, although in this case, skerries were likely colonized from nearby (<1 km) larger islands on which populations rapidly recovered following the algal bloom. Moreover, colonization seemed to occur by single females, in themselves constituting small founder groups carrying hundreds of embryos in their brood pouch that upon release in the new habitat provided a rapidly expanding new population. In addition, we have later found that females are extremely promiscuous and usually carry embryos sired by up to 20 males or more (Panova et al. 2010), adding considerable genetic variation to the founder group (Rafajlovic et al. 2013).

Dispersals of snail over very long distances are, obviously, extremely unlikely but must occur as *L. saxatilis* is found in Atlantic islands such as Iceland, Svalbard, The Faroe Islands and also has established a few remote populations in South Africa and the Mediterranean (Reid 1996). A paradox is that the closely related species, *L. littorea*, has not established populations in these remote sites despite a several week long larval stage and an otherwise largely overlapping distribution with *L. saxatilis* (Reid 1996). This made me curious about finding an explanation to the ‘Rockall paradox’, that is, the fact that the extremely remote and small (780 m^2^) Atlantic rock of Rockall (430 km north‐west Ireland) is only inhabited by species of macroalgae (with poor long‐distance dispersal) and a species of direct developing marine invertebrates (including *L. saxatilis*), while otherwise common intertidal species with long pelagic larval stages are completely lacking (Moore 1977). A likely explanation to the paradox is that while a planktonic larval stage may facilitate transport of single individuals to a remote spot, it will be hard to establish a local population as with broadcasting of larvae the offspring will dilute themselves to the extent that Allee effects will be a large problem. While a direct development will lower the chances of getting to a remote place, once there, a single fertilized female may efficiently establish a new population as all her offspring will remain in the local area (Johannesson 1988).

## Evolution of reproductive barriers

Returning to the evolution of reproductive barriers in contact zones of ecotypes, we abandoned the allozymes, as being partly under selection, and started to use putatively neutral markers (microsatellites) to estimate gene flow over contact zones. Using this approach, we found that gene flow was impeded over contact zones (*F*_ST_ values of 0.025 and 0.040) compared to differentiation over similar distances (~20 m) within ecotype populations either side of the contact zone (*F*_ST_ = 0.002–0.008) (Panova et al. 2006). Similar measurements performed over contact zones in Spain and the UK showed that gene flow over contact zones were 10–30% of gene flow within ecotypes (review by Johannesson et al. 2010a). A large part of this reduction (estimated from neutral markers) is likely explained by assortative mating of ecotypes observed in both laboratory and field (Johannesson et al. 1995b, 2008; Hollander et al. 2005). In addition, gene flow will be further impeded in loci and linked genomic regions that are under divergent selection (Johannesson and Tatarenkov 1997; Wilding et al. 2001; Mäkinen et al. 2008).

**Figure 5:**
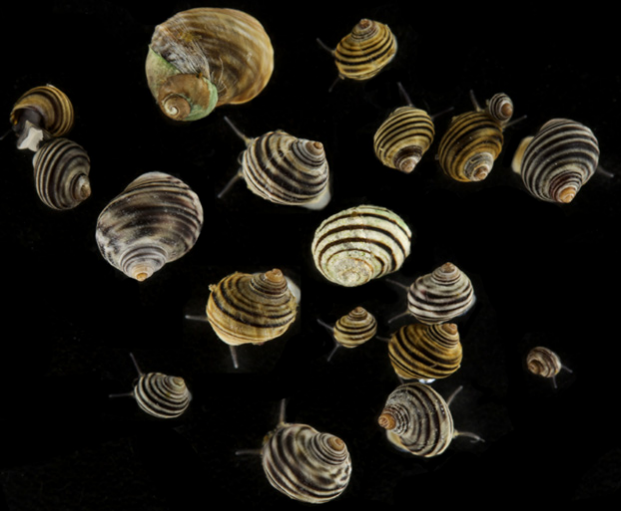
A family of full siblings produced by a cross between a Spanish *L. saxatilis* male of crab ecotype (the light and eroded snail in the middle) and a Swedish female of wave ecotype (the largest snail). For a mating to be successful, a very large wave ecotype and a small crab ecotype were deliberately chosen. Photo: Fredrik Pleijel.

As already stressed, isolation by distance is a component of fundamental importance to the genetic structure of the species. Nevertheless, individuals of the same ecotype from 150 km apart, mate as ‘happily’ with each other as with individuals from their local neighbourhood, and thus genetic differentiation accumulated due to isolation by distance do not *per se* contribute to a mating barrier at this scale of geographic separation (Hollander et al. 2005). Over even larger geographic distances (south‐west to north‐west Europe), gene flow cease completely and populations have been isolated for tens of thousands of years (Doellman et al. 2011; Panova et al. 2011). Notably, genomic separation between Spain and Sweden/UK is as large as the separation between *L. saxatilis* and its sibling species *L. arcana* (Panova et al. 2014). This raises the issue of genomic incompatibility that may have evolved between distant European populations of *L. saxatilis* due to isolation during thousands of years. In a small attempt to test this, a few Spanish crab ecotype males were crossed with virgin Swedish females of the wave ecotype. As they all produced fully vital and fertile offspring (Fig. 5), this suggest that there is no absolute intrinsic barrier to gene flow between populations representing both phenotypic and geographic extremes of the species.

## Parallel evolution and speciation under gene flow

**Figure 6:**
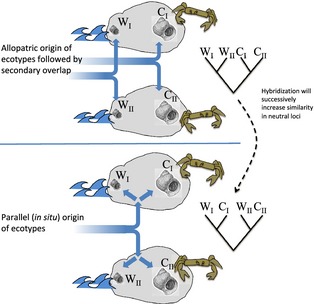
The two main hypotheses – allopatric origin and parallel origin of ecotypes. Although an allopatric origin of ecotypes should originally be obvious from the genetic relationship of samples, local hybridization will successively erase local differences in neutral markers and give rise to a similar pattern as for a parallel origin. However, using a Bayesian framework the expectations from the different models may be more comprehensively compared with empirical data (Butlin et al. 2014).

That the ecotypes occur in a repetitive pattern was obvious from the very first study (Janson 1982a), but the origin of the ecotypes was not easily sorted out. As outlined above, ecotype formation may be the result of divergence during an earlier allopatric stage followed by independent invasions of the two ecotypes into the same geographic areas and followed by hybridization in contact zones between microhabitats. The extreme alternative is that ecotypes are formed multiple times and in each local site, by differential selection. As a result of local divergence, reproductive barriers have evolved *in situ* in the face of gene flow, forming primary rather than secondary contact zones. While some (me included) enthusiastically supported the primary/sympatric model (Rolán‐Alvarez et al. 2004; Panova et al. 2006) others were more careful (Grahame et al. 2006). If all contact zones were indeed primary zones, this would challenge the strong belief that a sympatric origin of reproductive barriers was unlikely (Felsenstein 1981; Turelli et al. 2001; although see Feder et al. 1988; Bush 1994; Via 2001). Moreover, it would imply that evolution of ecotype differences and reproductive barriers between the crab and wave ecotypes occurred in a highly repetitive way, that is, being an example of parallel speciation (Schluter and Nagel 1995; Johannesson 2001). The two hypotheses – allopatric origin followed by secondary overlap and hybridization, and *in situ* and highly repetitive origin of primary zones – are unfortunately not easily separated. (Grahame et al. 2006; Johannesson et al. 2010a; Bierne et al. 2011, 2013). Repeated studies of ecotype divergence, using various markers and studying divergence in several different geographic areas, all showed that snails of different ecotypes from either side a contact zone were much more similar in neutral gene loci than were samples of the same ecotype from different islands or sites (Johannesson et al. 1993; Wilding et al. 2001; Rolán‐Alvarez et al. 2004; Grahame et al. 2006; Panova et al. 2006; Quesada et al. 2007). Despite these results, an early period of allopatry during which genetic differences in important loci were established, could not be rejected as extensive secondary hybridization may lead to a similar pattern (Fig. 6). A decisive suggestion from Roger Butlin was that we should compare comprehensive empirical data gathered in a similar way from all countries using the same approaches, with a more formal description of the two different hypotheses (old allopatric divergence of ecotypes followed by secondary overlap and gene flow, and parallel and local divergence) using an approximate Bayesian computation (ABC) approach (Beaumont 2010). For this study, 400 snails of crab and wave ecotypes were sampled in a hierarchical design over micro‐, regional‐ and national scales in Spain, the UK and Sweden. Using ABC modelling, we were able to more formally test the parallel evolution hypothesis against a hypothesis of allopatric divergence followed by secondary overlap and hybridization. The answer we got was, interestingly, a strong support for a parallel scenario and a weak support for an allopatric origin of ecotype differences both among and within geographic regions (Butlin et al. 2014). With earlier results (Panova et al. 2006; Quesada et al. 2007) suggesting parallelism also among islands or local sites, this suggested parallel evolution not only at among geographically distant sites but also at small, or even very small, spatial scales.

## A highly replicated system of divergences with gene flow

With local ecotypes and barriers to gene flow being formed *in situ,* the *L. saxatilis* system provides a large natural experiment with an outstanding level of replication at different levels of independence among populations. The molecular mechanisms of divergence may be compared both among close populations sharing a common gene pool (e.g. populations of small postglacial Swedish islands) and among populations of separate origins and gene pools representing divergence at a pan‐European scale.

This setting offers a unique opportunity to unveil, what mechanisms at the molecular level of evolution, promote the formation of local adaptation and reproductive barriers in the face of gene flow. Not least is there in this system opportunities to disentangle the role of deterministic and stochastic processes involved in speciation.

As outlined in Johannesson et al. (2010a), there are at least three possible mechanisms by which evolution of parallel phenotypes may occur: (i) Independent new mutations in the different sites may give rise to similar adaptive phenotypic changes. (ii) Similar phenotypic transitions (from one ecotype to another) may be the result of recombination and divergent selection from pre‐existing genetic variation (standing variation) of the first population established in a new site. (iii) Local phenotypic transitions are supported by the introduction of new favourable mutations, each of single origin but established and spread by selective sweeps resulting in ‘evolution in concert’ among populations of parallel sites. It may appear as evolution in concert will be weak in a system characterized by strong isolation by distance, but alleles favoured by selection will spread much more efficiently than neutral alleles (Morjan and Rieseberg 2004). Indeed, in the closely related species *Littorina fabalis*, a new allele of the arginine kinase locus has recently spread by a strong selective sweep throughout north‐west Europe (Kemppainen et al. 2011), and this species is also a direct developer with a population genetic structure similar to *L. saxatilis*, and a relatively poor gene flow. Also, in this species there is formation of ecotypes, and a small ecotype, specific to sheltered microhabitats of intertidal shores, is almost fixed for the new allele (Tatarenkov and Johannesson 1994, 1998, 1999).

## Exploring the details of speciation using genomic tools

To more fully understand the molecular mechanisms of divergence and how they link to ecological and stochastic drivers, genomewide sequencing technologies have become major tools. In a pioneering study, Craig Wilding, Roger Butlin and John Grahame used AFLP scans to screen genetic differentiation between crab (originally named ‘M’ in the UK) and wave (‘H’) ecotype populations in three sites in the UK (Wilding et al. 2001). With data from 306 AFLP loci, they found the same 15 loci to be consistently more different than expected by chance between wave and crab ecotypes in each of the three sites (maximum 41 km apart). Thus, 5% of all loci were outliers and highly differentiated between crab and wave ecotypes in each site, and the majority of these were also shared among sites. In a later study, using a BAC library, it was found that identified outlier fragments were representative of separate loci under selection, but they were located outside coding regions of the genome (Wood et al. 2008). In two loci, insertions of what seemed to be transposable elements upstream genes with putatively regulating functions were identified. Similar proportions of loci diverging between crab and wave ecotypes as AFLP outliers have also been found in Spanish and Swedish populations, but for some reason with much smaller proportions of shared outliers (Galindo et al. 2009; Hollander et al. 2015).

In parallel evolution, reuse of the same genetic variation from standing genetic variation is expected to be more frequent in more closely related populations than in highly independent populations. Thus, the expectation is that outlier loci between local pairs of crab‐wave snails would less often be the same ones (lower degree of sharing) as populations get increasingly independent (distant).

To investigate this, Anja Westram and colleagues compared the degree of sharing of crab‐wave outlier loci among three geographic regions, Spain, the UK and Sweden, and although populations are all geographically distant, the UK and Swedish populations have a more recent common ancestry while both are distantly related to the Spanish populations (Panova et al. 2011). Sequencing was in this study targeting the coding part of the genome (the transcriptome), and the results showed that overall sharing was similar and unexpectedly low (although higher than expected by chance), with most outlier loci being unique to populations from one country (Westram et al. 2014). Overall, 5–20% of all outlier loci were shared between any two countries and only very few (<6) loci were shared among all three countries, out of a total of ~7000 loci. Notably, there was no tendency for a higher sharing between the British and Swedish populations than between any of these and the Spanish population. Possibly, ecological differences among the three countries outside the crab‐wave dichotomy may contribute to the low reuse of genetic variation, in addition to local mutations being available at these large distances. In a recent study, we tried to eliminate the availability of area‐unique mutations and minimize idiosyncratic ecological differences among sites by analysing outliers found in ecotype pairs from populations within a small area (10 km). We sequenced a high number of random markers (RAD) throughout the genome and found that the proportion of sharing of outlier loci was still very low; 11–20% were shared between at least two islands and only 2–7% were shared among all three islands (M. Ravinet, A. Westram, K. Johannesson, R.K. Butlin, C. André and M. Panova, in submission). Notably, these proportions were similar to what had been found in an earlier comparison of two island populations in the same region using >1000 AFLP markers (Hollander et al. 2015).

There are various possible explanations for these observations. These results may suggest that selection is not so strong in most loci and therefore *F*_ST_ values will not consistently signal outlier status when compared over different population pairs. Alternatively, there are many different metabolic pathways, each involving alternative loci that lead to similar phenotypic divergences in the many quantitative traits that distinguish the ecotypes. If so, this would allow for idiosyncratic patterns of genetic divergence in each local population (Westram et al. 2014).

There is hope to at least partly resolve these issues by resequencing the complete genomes of crab and wave ecotypes which is now in progress (R. K. Butlin, K. Johannesson et al.) and supported by a draft *L. saxatilis* reference genome for a Swedish crab ecotype male (the IMAGO initiative led by A. Blomberg and K. Johannesson, www.cemeb.science.gu.se). Furthermore, comprehensive phenotypic and genetic data are being assembled from a number of contact zones in the Swedish archipelago in a new project led by Roger Butlin and Anja Westram, with the aim of detailed mapping of clines and selection coefficients for individual loci. In this project, we will also map genomic regions underlying quantitative traits both using admixture analysis of snails from contact zones and laboratory‐raised families of crab × wave crosses. This will generate information on phenotype–genotype links and the genomic architecture of divergence. In addition, the vast amounts of sequence data that are now being generated will also be useful in coalescence‐based modelling to test specific hypotheses of mechanisms of genomic divergence (work led by Marina Rafajlovic and Bernhard Mehlig). In individual genes already shown to be under strong selection (such as the *Aat*‐gene) evidence for the role of nonsynonymous mutations resulting in amino acid substitutions supports strong differential selection between the two microhabitats in some specific genes (M. Panova, M. Duvetorp, K. Johannesson, work in progress).

## Will ecotypes evolve into species?

Ecotypes appear to be reproductively isolated with a gene flow that is reduced to 10–30% of background gene flow over contact zones, but will the reproductive isolation ever evolve to a complete barrier and ecotypes become independent species? In collaboration with Sergey Gavrilet's laboratory, we used a spatially explicit, individual‐based model to investigate the timescale, driving forces and possible long‐term outcomes of the Swedish *L. saxatilis* populations (Sadedin et al. 2009). An important remark here is that, at least in Sweden, the ecotypes live in a postglacial environment and many islands have appeared as intertidal habitats only a few thousand years ago, and so the system, as a whole, is very young. Consequently, the system may not yet have reached an ‘equilibrium’, and modelling could be a way to predict long‐term outcomes and test the importance of different drivers.

Although, speciation was one possible outcome, many of our modelling scenarios stabilized in ecotype‐contact zone stages. One important reason for this seemed to be the bounded hybrid superiority, that is, hybrids being slightly more fit in the contact zone than are the parental forms, as observed in field experiments in both Sweden and Spain (Janson 1983a; Rolán‐Alvarez et al. 1997). According to the modelling results, hybrid superiority in the contact zone promotes the first step of speciation, that is ecotype formation, by supporting the evolution of a primary contact zone starting from one ecotype and successively expanding the population over the contact zone into the other microhabitat (a Swedish contact zone is illustrated in Fig. 3). However, bounded hybrid superiority also impedes completion of speciation by supporting a continued gene flow over the contact zone (Sadedin et al. 2009). A second factor that from the modelling approach appeared as important for the outcome was the mating pattern, although the relationship between mating system and the outcome of the modelling was rather complex, increased mating discrimination tended to increase genetic divergence of the two ecotypes.

From mate‐choice studies, there seem to be a strong relationship between size and mating preference, not only in *L. saxatilis* (Hollander et al. 2005; Johannesson et al. 2008) but as well in other littorinid species (e.g. Erlandsson and Johannesson 1994; Saltin et al. 2013). Size is also under strong divergent selection, as growth rate of the two ecotypes are genetically different (Janson 1982b; Johannesson et al. 1997). Size is hence a multi‐effect trait (‘a magic trait’) and a simple hypothesis may be that whenever divergent natural selection on size makes the two ecotypes different enough, speciation will be completed. More likely, however, is that speciation will be completed by the additive effect of divergence of several traits, some under direct divergent selection but also others that in various ways contribute to reinforce barriers that are already present. The more closely these traits are associated to each other in ‘trait‐association chains’, the more likely it will be that speciation will evolve to completion (Smadja and Butlin 2011).

Notably, the Spanish system of crab and wave ecotypes is likely much older than both the Swedish and British systems (Panova et al. 2011), but despite different ages, and different extensions of the contact zones – small ‘vertical’ contact zones in Sweden, but extensive ‘horizontal’ contact zones in UK and Spain – all three systems are quite similar in terms of size dimorphism of ecotypes and also in strength of the reproductive barriers (Johannesson et al. 2010a; Butlin et al. 2014; Westram et al. 2014). This, perhaps, corroborate the suggestion that the size dimorphism caused by divergent natural selection is after all the most crucial component to the strength of the reproductive barrier.

## What have we learnt from the snail system?

Rapid formation of ecotypes is more or less expected under strong divergent selection in a system with restricted gene flow and the *Littorina* system is a clear‐cut empirical example of this. Notably, ecotype formation is facilitated with hybrids being rather fit in the hybrid zone, but ecotype formation is not the same thing as speciation, and factors favouring ecotype formation may be detrimental to speciation (Sadedin et al. 2009).

In the *Littorina* system, we now have strong support for the evolution of reproductive barriers in parallel and under gene flow. Indeed, this is probably the first time that a model of sympatric divergence (primary contact zone) has been formally tested and gained support over the alternative model of old allopatric divergence followed by secondary overlap and hybridization (Butlin et al. 2014). Although, we are just at the doorstep of disentangling the molecular mechanisms behind the parallel formation of primary contact zones, available ecological information will help targeting the important traits. For example, it seems clear that snail size is both a trait under strong divergence selection and a trait important to mate choice, with strong nonrandom mating in the hybrid zone (Johannesson et al. 1995b), as well as in laboratory experiments (Hollander et al. 2005). Thus, size seems to be a ‘magic’ or multi‐effect trait, and this likely enhances the formation of barriers between the ecotypes (Servedio et al. 2011; Smadja and Butlin 2011). In addition, it seems as there is, in many littorinid species, a strong tendency in males in general to mate females that are larger than themselves (e.g. Erlandsson and Johannesson 1994). Possibly, such a trait is coded by the same allele (or the same alleles) in all populations and favoured both inside and outside contact zones. If so, this would escape the problem of recombination in the contact zone that breaks up associations of traits involved in barriers (Smadja and Butlin 2011), which has for long been considered a main obstacle to formation of barriers under gene flow (Felsenstein 1981).

It is truly fascinating that under similar types of divergent selection, more or less the same phenotypes may evolve repeatedly along with similarly strong reproductive barriers (Butlin et al. 2014), while within phenotypes, there seems to be no barriers to gene flow other than geographic isolation. Disentangling the genetic architecture of gene flow barriers is an ambitious, but very important task, and the few results already emerging suggest surprisingly low levels of sharing of the same genetic variation involved in barriers of even very closely related populations (e.g. Westram et al. 2014; Hollander et al. 2015; M. Ravinet, A. Westram, K. Johannesson, R. K. Butlin, C. André and M. Panova in submission). This is very surprising and may suggest a role for stochastic processes also in speciation assumed to be largely driven by ecological divergence. Another surprising observation in the *Littorina* system is the strong reproductive isolation that has evolved in what seems to be a complete absence of reinforcement, which is usually consider as a strong driving force in speciation (Turelli et al. 2001; Ortiz‐Barrientos et al. 2009). In *L. saxatilis,* hybrids have a high fitness in the contact zone, avoiding hybridization in the contact zone has therefore no direct benefits to an individual, and hence selection promoting further prezygotic isolation may be small or absent.

However, multiple barriers to gene flow are established along the genome due to divergent selection over contact zones, and an interesting issue is if there is any mechanism that tend to increase the association of these barriers and in this way successively strengthen the overall barrier to gene flow (Smadja and Butlin 2011; Yeaman and Whitlock 2011; Nosil and Feder 2012), and by this speciation may finally be completed.

It is my hope, that a comprehensive exploration of the highly replicated formation of reproductive isolation in the face of gene flow in the *Littorina* system, will eventually contribute a lot to our understanding of how barriers to gene flow are established and how they evolve, and in particular, under what circumstances they grow until gene flow has ceased and speciation is completed.
